# Corneal confocal microscopy is a rapid reproducible ophthalmic technique for quantifying corneal nerve abnormalities

**DOI:** 10.1371/journal.pone.0183040

**Published:** 2017-08-17

**Authors:** Alise Kalteniece, Maryam Ferdousi, Safwaan Adam, Jonathan Schofield, Shazli Azmi, Ioannis Petropoulos, Handrean Soran, Rayaz A. Malik

**Affiliations:** 1 Institute of Cardiovascular Sciences, Cardiac Centre, Faculty of Medical and Human Sciences, University of Manchester and NIHR/Wellcome Trust Clinical Research Facility, Manchester, United Kingdom; 2 Weill Cornell Medicine-Qatar, Research Division, Qatar Foundation, Education City, Doha, Qatar; Bascom Palmer Eye Institute, UNITED STATES

## Abstract

**Purpose:**

To assess the effect of applying a protocol for image selection and the number of images required for adequate quantification of corneal nerve pathology using in vivo corneal confocal microscopy (IVCCM).

**Methods:**

IVCCM was performed in 35 participants by a single examiner. For each participant, 4 observers used a standardized protocol to select 6 central corneal nerve images to assess the inter-observer variability. Furthermore, images were selected by a single observer on two occasions to assess intra-observer variability and the effect of sample size was assessed by comparing 6 with 12 images. Corneal nerve fiber density (CNFD), branch density (CNBD) and length (CNFL) were quantified using fully automated software. The data were compared using the intra class correlation coefficient (ICC) and Bland-Altman agreement plots for all experiments.

**Results:**

The ICC values for CNFD, CNBD and CNFL were 0.93 (P<0.0001), 0.96 (P<0.0001) and 0.95 (P<0.0001) for inter-observer variability and 0.95 (P<0.0001), 0.97 (P<0.001) and 0.97 (P<0.0001) for intra-observer variability. For sample size variability, ICC values were 0.94 (P<0.0001), 0.95 (P<0.0001), and 0.96 (P<0.0001) for CNFD, CNBD and CNFL. Bland-Altman plots showed excellent agreement for all parameters.

**Conclusions:**

This study shows that implementing a standardized protocol to select IVCCM images results in high intra and inter-observer reproducibility for all corneal nerve parameters and 6 images are adequate for analysis. IVCCM could therefore be deployed in large multicenter clinical trials with confidence.

## Introduction

The use of corneal confocal microscopy (CCM) for the assessment of neuropathy has grown considerably over the last decade. Many studies have shown corneal nerve pathology in patients with a range of peripheral neuropathies [1–7]. Indeed, the assessment of corneal nerve morphology using CCM has been suggested as a surrogate end point in the assessment of diabetic peripheral neuropathy [8]. Several morphological parameters including corneal nerve fiber length (CNFL), corneal nerve fiber density (CNFD) and corneal nerve branch density (CNBD) have been used to quantify sub basal corneal nerve pathology [9–11]. Previous studies have evaluated the reproducibility of measuring these parameters in the assessment of neuropathy [12–15]. Manual quantification of CNFL in patients with type 2 diabetes was shown to be highly repeatable, but a more experienced observer enhanced the accuracy of analysis [12]. Petropoulos et. al. [13] demonstrated excellent inter and intra observer repeatability of manual CNFD and CNFL but not CNBD quantification, indicating the importance of expert assessment to ensure optimal identification of nerve branches or the need for automated analysis.

Vagenas et al. [16] have shown that corneal nerve quantification requires the assessment of 5 to 8 central corneal images, where the sub-basal nerve plexus is most dense and most previous CCM studies have examined the central corneal area [17]. However, even slight saccadic eye or device movement can result in images being acquired from a non-central area, which can potentially impact on the results, particularly in longitudinal and interventional studies. Thus the examiners have to choose 5–8 optimal images from more than 100 images taken during the examination, which can be challenging, especially as the quality of the images can be quite variable. Different studies use different protocols for selecting the number and type of images used for quantification of corneal nerve parameters. There are very few studies, which have assessed whether or not these protocols are reproducible [14]. The purpose of this study was to establish the intra and inter-rater reproducibility of a standardized protocol for image selection and to define the optimal image sample size, which could be deployed in multicenter studies.

## Methodology

### Study subjects

Images from 35 randomly selected participants with obesity who had previously undergone detailed assessment of neuropathy and corneal confocal microscopy were included. Informed written consent was obtained from all patients before their participation in the study. This research adhered to the tenants of the declaration of Helsinki and was approved by Greater Manchester Central and North Manchester research ethic committees. Participants were excluded if they had corneal dystrophy or cystic corneal disorders.

### Corneal confocal microscopy

Corneal confocal microscopy (CCM) was performed in both eyes of participants using a Heidelberg Retinal Tomograph III with Rostock Cornea Module (HRT III RCM) (Heidelberg Engineering GmbH, Heidelberg, Germany) according to our previously established protocol [18]. The illumination source utilised in this model was a 670nm diode laser classified as a class 1 laser system. The laser beam spot was 1μm in diameter and the instrument field of view was 400 x 400μm with a 63x objective lens. Optical resolution is 10μm/pixel with 2D digital image size being 384 x 384 pixels. Live imaging of the cornea by a CCD camera attached to the microscope determined the location of examination. All CCM examinations were performed by an expert examiner (OB1) and images were obtained from the central cornea at the level of sub basal nerve plexus using the section mode.

### Image selection

To evaluate the inter observer variability when selecting CCM images; four independent observers from the University of Manchester, United Kingdom participated in the study. Observer 1 (OB1) and observer 2 (OB2) were experienced optometrists in identifying and extracting CCM images and considered ‘experts’. Observers 3 and 4 (OB3 and OB4) were medical doctors without any experience of working with CCM and had minimal experience in selecting and analyzing images of the corneal sub-basal nerve plexus and were therefore considered ‘beginners’. All four observers’ independently selected six images of the sub-basal nerve plexus from the central cornea (3 per eye) for each subject and followed a standardized protocol detailed below (S1 Fig).

**Fig 1:**
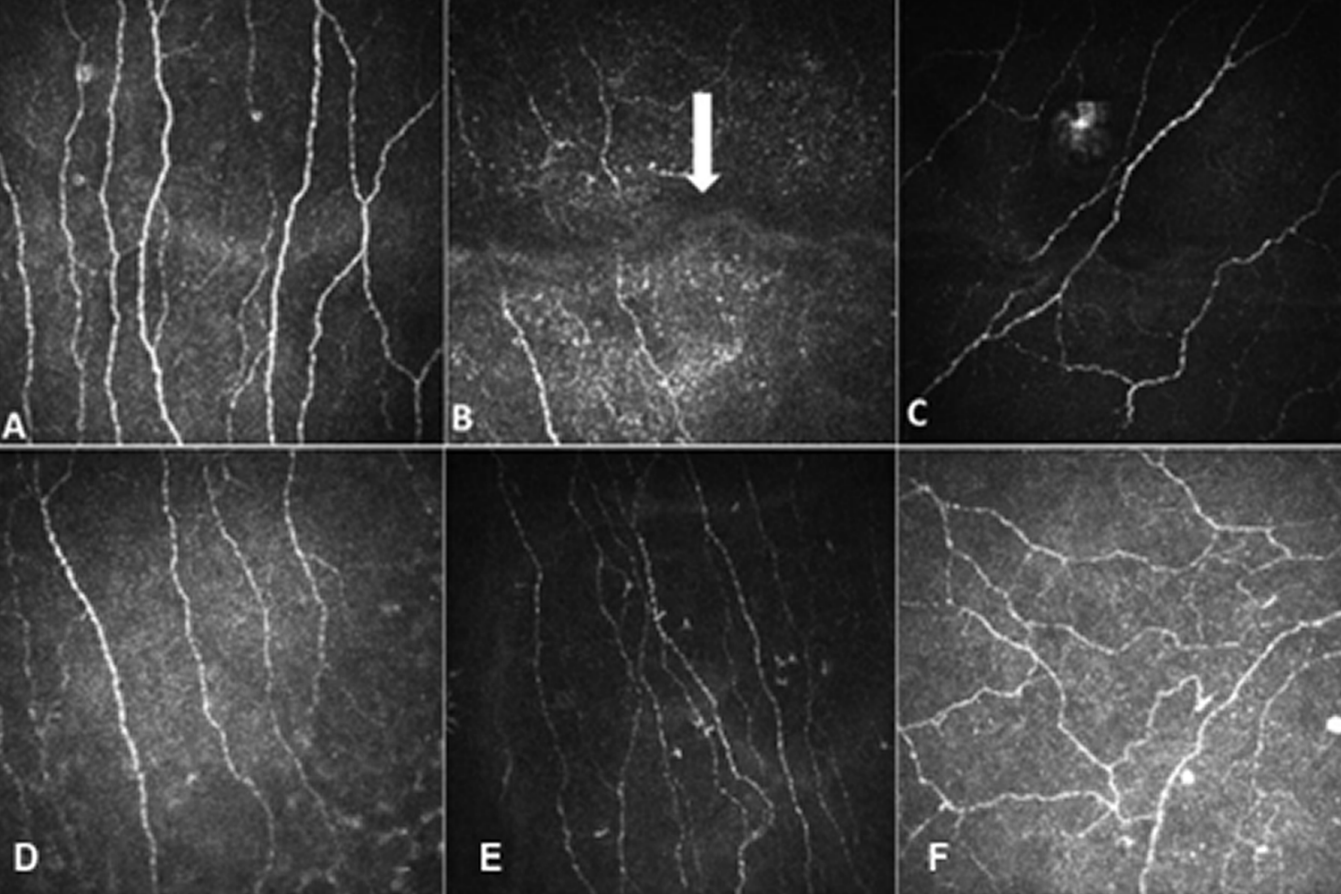
Examples of CCM images. A) a high quality CCM image of sub basal nerves from the central cornea (nerves are vertical); B) A CCM image with a pressure line (indicated with an arrow); C) A CCM image of sub basal nerves from the peripheral cornea (oblique nerves); D) a CCM image with overlapping corneal layers (stroma and sub-basal nerve plexus) as can be seen with the keratocytes in the inferior and peripheral zone; E) a low contrast CCM image; F) inferior whorl of the cornea.

- Selected images were required to be from the center of the cornea. Image choice from the central cornea was based on both the live image on the CCD camera as well as the orientation of corneal nerves. The red reflex, (the reflex of the laser beam on the cornea), was required to be in the center of the eye in the live image for the selected image. With regard to the orientation of the nerves they are usually vertical in the center and become oblique in the peripheral cornea, therefore only images with vertically oriented nerves were selected.
- Images of differing numbers of main nerves were selected such that images with the least, intermediate and highest number of main nerves were selected. This is especially relevant when images are selected from control subjects with many nerves, compared to patients with severe neuropathy with very few nerves.
- Images were required to be of high quality with paucity of pressure lines, optimal contrast and with an absence of overlap, and where part of the image was from either the epithelial or stromal layers in addition to the sub-basal layer (Fig 1). Fig 1, A is an example of a selected image fulfilling all the criteria for image selection and Fig 1, B-F are examples of images that do not meet the required criteria and should not be selected.

To evaluate the intra-observer variability; OB2 exported 6 images per participant on two separate occasions (OB2a and OB2b) with a one-week interval in between (S2 Fig).

To evaluate whether the image sample size affects corneal nerve quantification, OB1 exported 6 images on one occasion (OB1a) and then 12 images on a separate occasion (OB1b) for each participant.

### Image analysis

Images were analyzed using purpose designed fully automated nerve analysis software (Corneal Nerve Fiber Analyser V.2) (ACCMetrics) (University of Manchester, Manchester, United Kingdom) [19] to quantify corneal nerve fibre density (CNFD–a total number of main nerves per square millimeter) (no./mm^2^), corneal nerve fibre length (CNFL–a total length of main nerves and nerve branches per square millimeter) (mm/mm^2^) and corneal nerve branch density (CNBD–a total number of main nerve branches per square millimeter) (no./mm^2^).

### Statistical analysis

Statistical analysis was performed using IBM SPSS v19.0 (Chicago, IL, USA). The Shapiro-Wilk normality test was employed to assess whether the data is normally distributed or not. Paired sample t test and repeated measure ANOVA was used to assess the differences between measurements. Intraclass correlation coefficient (ICC) was measured to identify inter and intra-observer repeatability and were considered as excellent if 0.80–1.0. The Bland-Altman agreement plots [20] were used to assess the agreement between two measurements. All data are presented as mean ± SD. The data used for statistical analysis in this study are fully available on “https://figshare.com/s/125f0c92e3535bcc8f5f“.

## Results

### Demographic data

Thirty-five subjects (18 men and 17 women) with diabetes and/ or obesity were included in this study. The average age was 49.97 ± 12.47 years, HbA1c was 47.77 ± 21.35 mmol/mol, BMI was 41.19 ± 15.24 kg/m^2^ and the neuropathy disability score (NDS) was 2.12 ± 2.78.

### Inter-observer reproducibility

Table 1 shows the Mean±SD of all CCM parameters measured by each independent observer. CNFD (P = 0.3), CNBD (P = 0.2) and CNFL (P = 0.3) were not significantly different among all observers.

**Table 1:** Corneal nerve parameters expressed as mean ±SD for each observer with significant difference and ICC value with significance.

	OB1a	OB2a	OB3	OB4	P value	ICC VALUE
**CNFD (no./mm**^**2**^**)**	24.59 ±4.59	25.25 ± 6.26	24.97 ± 6.28	24.87 ± 5.78	0.3	r = 0.93, P<0.0001
**CNBD (no./mm**^**2**^**)**	32.51 ±2.51 n	35.27 ± 16.05	33.63 ± 15.57	33.25 ± 15.08	0.2	r = 0.96, P <0.0001
**CNFL (mm/mm**^**2**^**)**	14.81 ±4.81	14.97 ± 2.91	14.86 ± 2.91	14.44 ± 2.65	0.3	r = 0.95, P <0.0001

**Fig 2:**
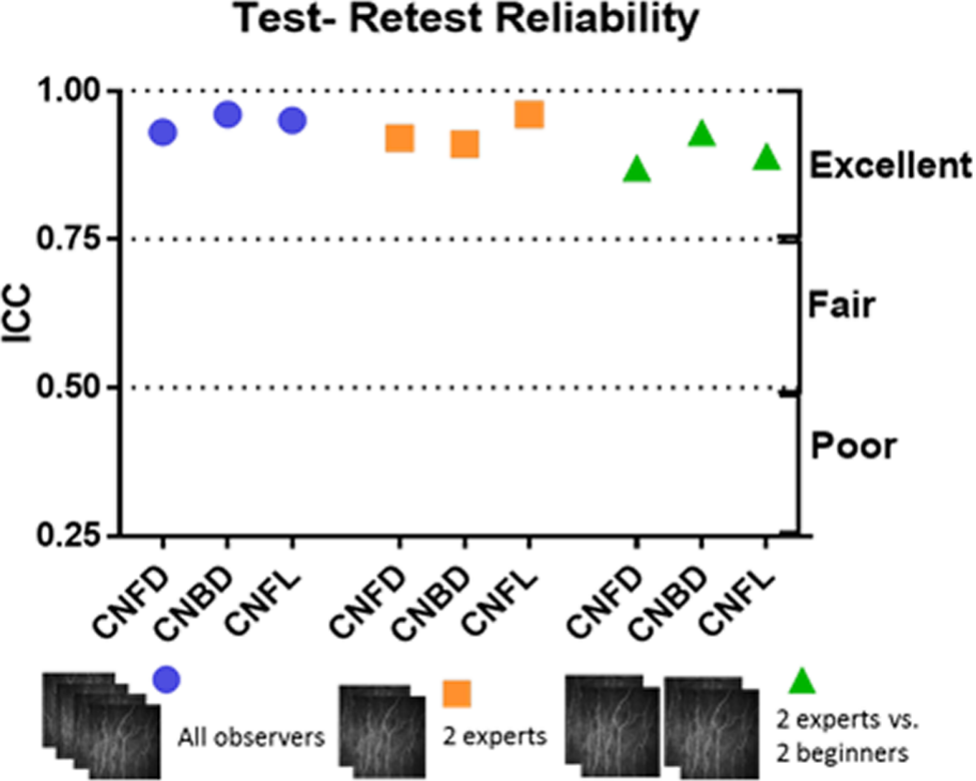
Summary plot of ICC values for all experiments in relation to reliability of assessment of CCM parameters.

Among 4 observers (OB1a, OB2a, OB3, OB4) the ICC values with confidence intervals (CI) were 0.93 (95% CI- 0.93–0.97) for CNFD, 0.96 (95% CI-0.95–0.98) for CNBD and 0.95 (95% CIs-0.95–0.98) for CNFL (Table 1). The ICC values between both experts (OB1 and OB2) were 0.92 (95% CI-0.85–0.96) for CNFD, 0.91 (95% CI-0.82–0.95) for CNBD and 0.96 (95% CI 0.92–0.98) for CNFL. Fig 2 illustrates the ICC values in relation to reliability of assessment of CCM parameters.

**Fig 3:**
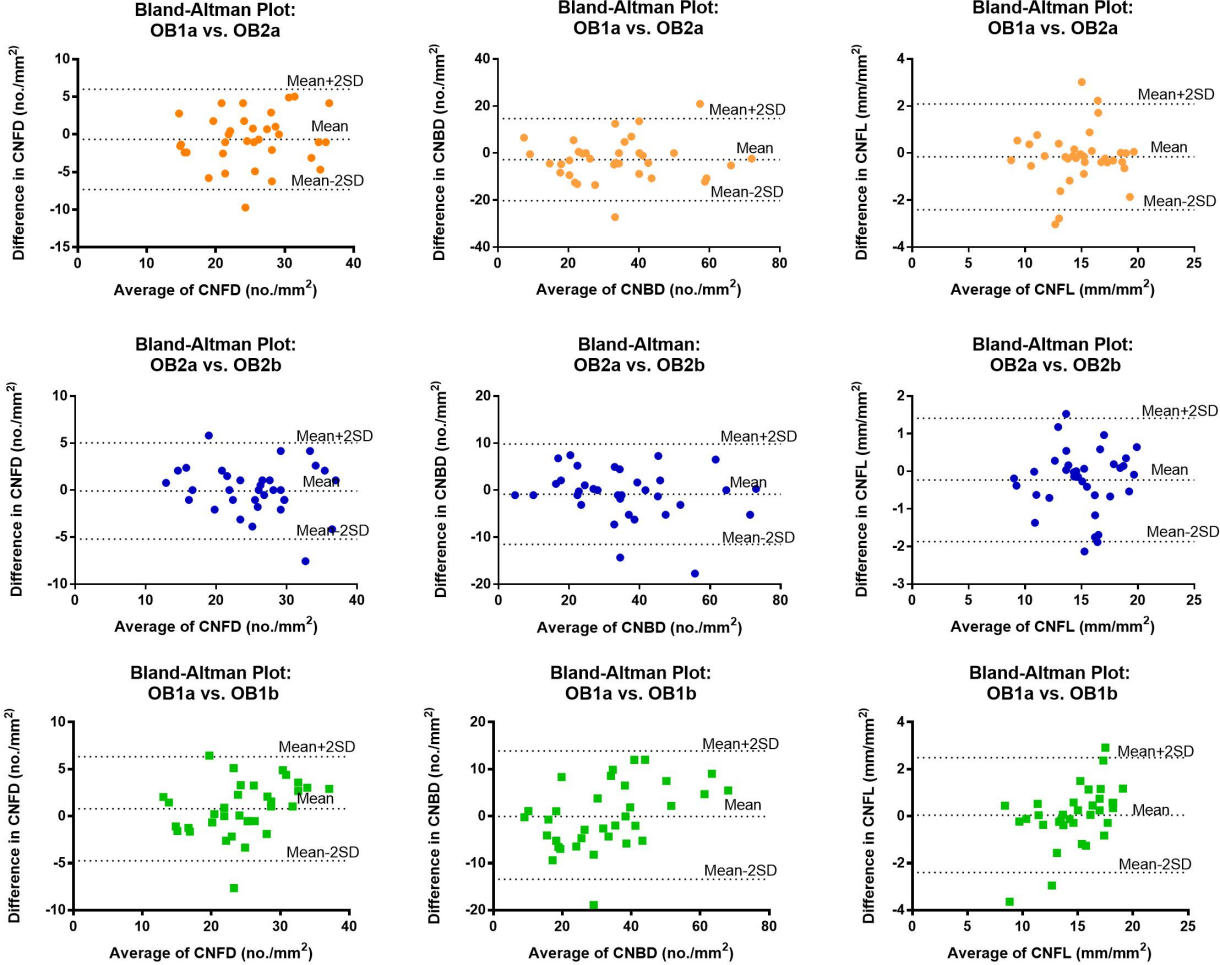
**Bland-Altman agreement plots;** first row, inter-observer reliability (OB1a and OB2a) for CNFD (A), CNBD (B) and CNFL (C); second row, Intra-observer repeatability (OB2a vs OB2b) for CNFD (D), CNBD (E), CNFL (F) and sample size validity (OB1a and OB1b) for CNFD (G), CNBD (H) and CNFL (I).

Fig 3 shows the Bland-Altman agreement plots for CCM parameters measured by observer 1 and observer 2 (OB1a vs OB2a). The mean differences (± SD) between both experts were 0.65 ± 3.4/mm^2^ for CNFD, 2.75 ± 8.9/mm^2^ for CNBD and 0.16 ± 1.15/mm^2^ for CNFL. ICC values and mean difference of CCM parameters among all observers are shown in Table 2.

**Table 2:** Intra class correlation coefficient (ICC) and the mean± SD of differences between different observers for corneal nerve fibre density (CNFD), corneal nerve branch density (CNBD) and corneal nerve fibre length (CNFL).

		OB2a	OB3	OB4
**CNFD(no./mm**^**2**^**)**	OB1a	ICC = 0.92, p< 0.0001 Mean difference:0.65±3.40	ICC = 0.89, p< 0.0001 Mean difference:0.37±3.99	ICC = 0.85, p< 0.0001 Mean difference:0.27±4.47
	OB2a		ICC = 0.91, p< 0.0001 Mean difference: 0.28±3.59	ICC = 0.83, p< 0.0001 Mean difference: 0.28±3.59
	OB3	ICC = 0.91, p< 0.0001 Mean difference:0.28±3.59		ICC = 0.84, p< 0.0001 Mean difference:0.10±4.53
	OB4	ICC = 0.83, p< 0.0001 Mean difference:0.38±4.57	ICC = 0.84, p< 0.0001 Mean difference:0.10±4.53	
**CNBD(no./mm**^**2**^**)**	OB1a	ICC = 0.91, p< 0.0001 Mean difference:2.75±8.90	ICC = 0.91, p< 0.0001 Mean difference:1.11±8.82	ICC = 0.94, p< 0.0001 Mean difference:0.73±7.33
	OB2a		ICC = 0.94, p< 0.0001 Mean difference: 1.64±0.03	ICC = 0.92, p< 0.0001 Mean difference: 2.02±8.14
	OB3	ICC = 0.94, p< 0.0001 Mean difference:1.64±0.03		ICC = 0.95, p< 0.0001 Mean difference:0.38±6.60
	OB4	ICC = 0.92, p< 0.0001 Mean difference:2.02±8.14	ICC = 0.95, p< 0.0001 Mean difference:0.38±6.60	
**CNFL(mm/mm**^**2**^**)**	OB1a	ICC = 0.96, p< 0.0001 Mean difference:0.16±1.14	ICC = 0.93, p< 0.0001 Mean difference:0.04±1.49	ICC = 0.88, p< 0.0001 Mean difference:0.36±1.76
	OB2a		ICC = 0.93, p< 0.0001 Mean difference: 0.11±1.44	ICC = 0.88, p< 0.0001 Mean difference: 0.52±1.74
	OB3	ICC = 0.93, p< 0.0001 Mean difference:0.11±1.44		ICC = 0.90, p< 0.0001 Mean difference: 0.41±1.62
	OB4	ICC = 0.88, p< 0.0001 Mean difference:0.52±1.74	ICC = 0.90, p< 0.0001 Mean difference:0.41±1.62	

### Intra-observer repeatability

There was no significant difference between CNFD (25.25±6.25 vs. 25.34±6.58, P = 0.8), CNBD (35.27±16.05 vs. 36.12±17.23, P = 0.3) and CNFL (14.97±2.91 vs. 15.20±2.88, P = 0.1) for an expert on the first and second occasion (OB2a and OB2b), respectively. The ICC values for OB2 on both occasions were 0.95 (95% CI-0.91–0.97) for CNFD, 0.97 (95% CI-0.94–0.98) for CNBD and 0.97 (95% CI-0.95–0.98) for CNFL (Fig 1). Fig 3A–3C shows Bland-Altman agreement plots on both occasions (OB2a and OB2b) to appreciate the intra-observer repeatability. The mean differences (± SD) for OB2 on both occasions of assessment were 0.93 ± 2.6 for CNFD, 0.85 ± 5.4 for CNBD and 0.23 ± 0.8 for CNFL.

### Sample size variability

To measure the optimal image sample size, CNFD, CNBD and CNFL were measured by OB1 on two separate occasions (6 images on the first occasion and 12 images on the second occasion). CNFD (24.50±6.61 vs. 23.81±5.71, P = 0.1), CNBD (32.51±16.46 vs. 32.50±13.53, P = 0.9) and CNFL (14.80±2.96 vs. 14.62±2.60, P = 0.3) did not differ significantly on the first and second occasions. ICC value between OB1a and OB1b were 0.94 (95% CI-0.88–0.97) for CNFD, 0.95 (95% CI-0.89–0.97) for CNBD and 0.96 (95% CI-0.92–0.98) for CNFL. Fig 3D–3F) shows the Bland-Altman agreement plots for OB1 on two separate occasions (OB1a and OB1b). The mean differences (± SD) for OB1 between both occasions were 0.78 ± 2.79 for CNFD, 0.01 ± 6.87 for CNBD and 0.18 ± 1.07 for CNFL.

## Discussion

The quantification of corneal sub-basal nerve morphology has evolved to become an important technique for assessing the severity and progression of diabetic neuropathy [3, 4, 8, 10, 17]. Several studies have defined the reproducibility of CCM for the assessment of diabetic neuropathy [12, 13, 15, 21]. These studies primarily evaluated the variability of manual and automated analysis of CCM images by different observers. However, a key determinant of what one quantifies is the images one selects for the quantification. This is especially relevant if CCM is to be deployed in multicenter studies and in longitudinal or intervention studies, when repeat quantification is required. In the present study we have removed the influence of nerve fibre recognition error by deploying automated image analysis. Based on our experience with CCM we developed a protocol that takes into account the key factors that will influence the quantification of corneal nerve parameters.

This is the first study showing the utility and reproducibility of a standardized protocol for image selection. We demonstrate excellent inter- and intra-observer concordance for each nerve fibre metric. The highest intra-observer and inter-observer concordance was found for CNFL and the highest inter-observer concordance amongst all observers was found for corneal nerve branch density (CNBD). Previously Hertz. et.al. (2011) have concluded that CNFL is the best biomarker to identify patients with and at risk of sensorimotor polyneuropathy, whereas Petropoulos et.al. showed that corneal nerve fibre density is the most repeatable corneal nerve parameter [13, 21]. Certainly in the present study the inter- and intra-observer repeatability was maximal for CNFL. Ostrovski et al. have recently shown that corneal nerve parameters differ significantly when using two different protocols; a semi-automated (2 images) and fully automated (all captured images) protocol for the quantification of corneal nerve parameters [14]. In contrast with Ostrovski et al., our study shows that by following a standardized protocol, corneal nerve parameters did not differ significantly when analyzing 6 images or 12 images. Therefore, and as previously suggested by Vagenas et al. (2012), exporting and analyzing 6 images from central corneal sub basal nerves would be sufficient for the assessment of neuropathy [16].

Although intra class correlation was excellent for both experiments comparing the results measured by both experts and 2 experts versus 2 beginners, the mean of the difference was lower when comparing the results between 2 experts. This suggests that the level of expertise will affect the test retest variability as previously suggested by Petropoulos et al 2013 [13]. In addition to the level of expertise, another important factor is the density of corneal nerves. The Bland-Altman agreement plots indicate that the difference between CCM parameters measured by two observers was higher for subjects with higher and lower density of corneal sub basal nerve.

In conclusion, we show that the application of a standardized method for selecting and analyzing images of the corneal sub-basal plexus with automated quantification provides excellent inter and intra observer repeatability irrespective of investigator experience and image number.

## Supporting information

S1 Fig First row, 6 CCM images from central sub basal nerve plexus exported by expert; second row, 6 CCM images from the same patients but exported by a beginner. (PDF)

S2 Fig First row, 6 CCM images from central sub basal nerve plexus exported by an expert (OB2a) in first occasion; second row, CCM images exported by the same examiner (OB2b) from the same patient in the second occasion. (PDF)
