# Supplementary material for: Corneal confocal microscopy is a rapid reproducible ophthalmic technique for quantifying corneal nerve abnormalities
Source: PLoS One. 2017 Aug 17;12(8):e0183040. doi: 10.1371/journal.pone.0183040 (PMC5560560; doi:10.1371/journal.pone.0183040)
Supplement: S1 Fig — (PDF) [file pone.0183040.s001.pdf]

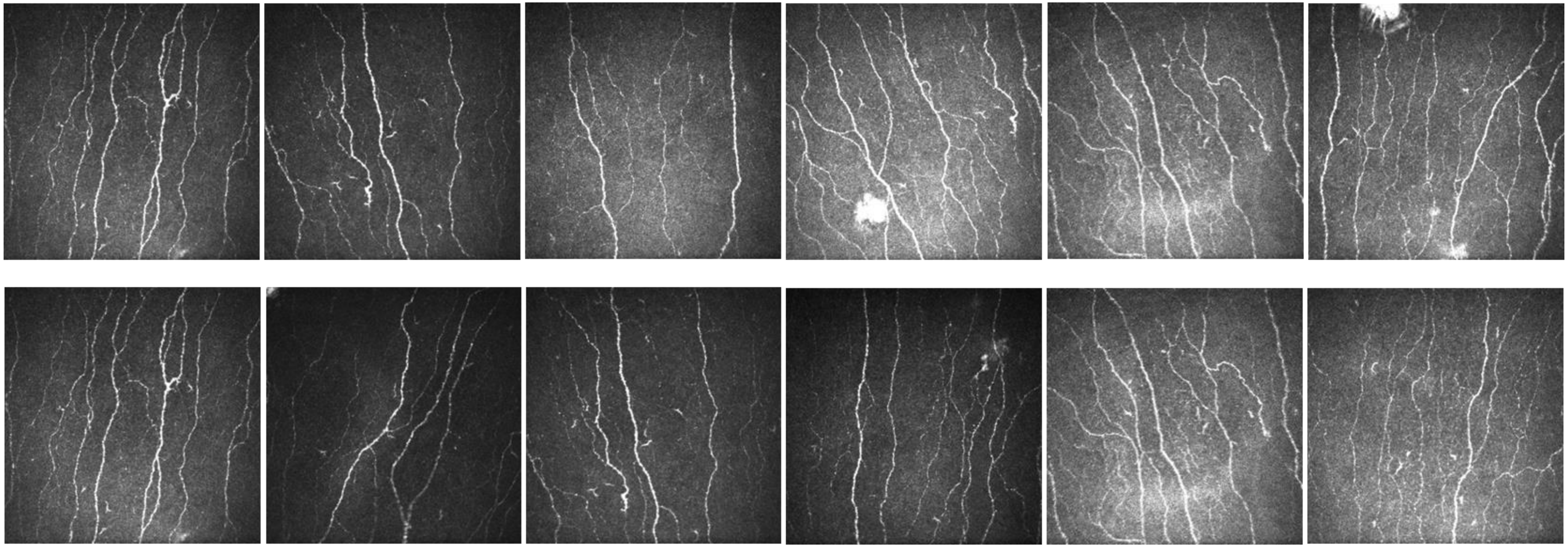

S1 figure: first row, 6 CCM images from central sub basal nerve plexus exported by expert; second row, 6 CCM images from the same patients but exported by a beginner.
