# Supplementary material for: Corneal confocal microscopy is a rapid reproducible ophthalmic technique for quantifying corneal nerve abnormalities
Source: PLoS One. 2017 Aug 17;12(8):e0183040. doi: 10.1371/journal.pone.0183040 (PMC5560560; doi:10.1371/journal.pone.0183040)
Supplement: S2 Fig — (PDF) [file pone.0183040.s002.pdf]

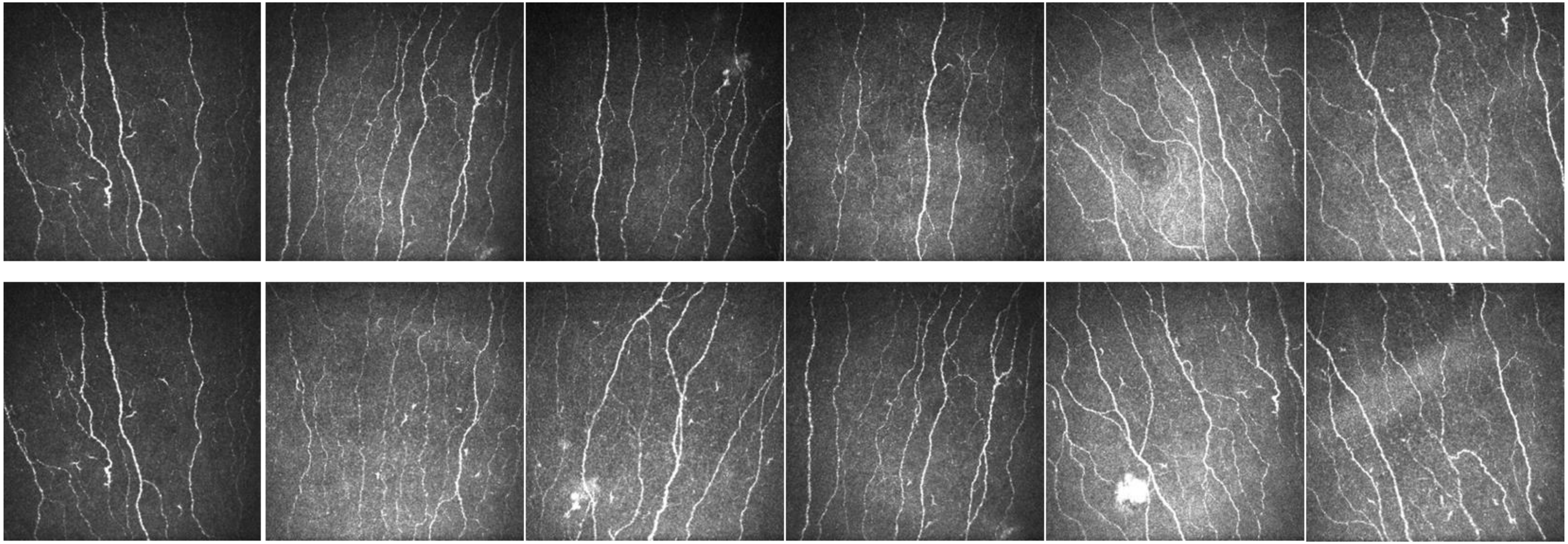

S2 figure: First row, 6 CCM images from central sub basal nerve plexus exported by an expert (OB2a) in first occasion; second row, CCM images exported by the same examiner (OB2b) from the same patient in the second occasion.
